# Understanding the social and cognitive influences on the adoption of COVID-19 non-pharmaceutical interventions: a survey of a Saudi Arabian sample

**DOI:** 10.3389/fpubh.2025.1588931

**Published:** 2025-06-27

**Authors:** Ahmed Alobaydullah, Andrew Scott LaJoie, Susann Denise Thomas, Raphael Fumey, Abdulrahman Alsaleem

**Affiliations:** ^1^Emergency Medical Services Program, College of Applied Medical Sciences, King Saud bin Abdulaziz University for Health Sciences, Al Ahsa, Saudi Arabia; ^2^King Abdullah International Medical Research Center, Al Ahsa, Saudi Arabia; ^3^Department of Health Promotion and Behavioral Sciences, University of Louisville, Louisville, KY, United States

**Keywords:** preventive behaviors, COVID-19, social cognitive theory, non-pharmaceutical interventions, social norms, Saudi Arabia

## Abstract

**Introduction:**

COVID-19 vaccines prevent death and severe illness, but not infection. Public health leaders continue to stress the importance of non-pharmaceutical intervention behaviors (NPIs). However, little is known about how social, environmental, and cognitive factors influence the adoption of NPIs.

**Methods:**

A theory-informed survey was distributed to adults living in Al-Ahsa, Saudi Arabia. The primary focus is self-reported adherence with NPIs. Socio-environmental factors included observational learning, social norms, and media. Personal cognitive factors included self-efficacy, outcome expectancies, and knowledge.

**Results:**

The mean age was 31 years (*n* = 368), most participants were males (62%), and the study sample were significantly more vaccinated (40%) than the Saudi public (5.3%). NPIs were strongly correlated with social norms (*r* = 0.73, *p* < 0.001), and positively correlated with self-efficacy (*r* = 0.25, *p* < 0.001). Females reported higher NPIs adherence rates (*M* = 17.04, *SD* = 3.86) than males (*M* = 16.29, *SD* = 4.12). The hierarchical multiple regression model revealed that socio-environmental factors explained a significant proportion of variance in NPIs (*R*^2^ = 0.52, *p* < 0.001).

**Discussion:**

The findings highlight the usefulness of a social cognitive model in predicting NPIs. The study shows that social factors, especially social norms, significantly influence the adoption of NPIs. Hence, health organizations should consider social factors when developing campaigns against current and future infectious diseases.

## 1 Introduction

Since December 2019, SARS-CoV-2 has spread throughout the world, posing health, social, and economic threats that are comparable to previous pandemics such as the Spanish Influenza of 1918. The current global prevalence of COVID-19 is estimated to be 777 million cases and over 7 million deaths (1). To many, this is considered a significant undercount of cases and fatalities (2). The social context of the current pandemic creates unique obstacles to controlling its spread, as world travel and international trade are much greater than in the past. Further, while anti-vaccine and anti-science attitudes have existed previously, the advent of social media has amplified the impact of false or misleading information (3, 4).

Initially, the lack of effective vaccines and medications forced health organizations such as the U. S. Centers for Disease Control and Prevention (CDC) and the World Health Organization (WHO) to rely on the use of Non-Pharmaceutical Interventions (NPIs) to attenuate the spread of COVID-19 (5, 6). Recommended NPIs include frequently washing hands with soap and warm water, wearing a mask or a face covering in public, keeping a physical distance of at least 6 feet in public, and self-quarantine when directed (5, 7). Without adequate medical technologies, health authorities resorted to controlling the pandemic through social and behavior change (6). In some countries, such as Saudi Arabia, NPIs recommendations were supplemented with government-imposed lockdowns and curfews (8).

Residents of Saudi Arabia have bitter experiences with infectious disease outbreaks; the Middle Eastern Respiratory Syndrome (MERS), which had a high fatality rate of 34% (9), resulted in 780 deaths in 7 years. The lessons learned from MERS prepared the Saudi healthcare system to respond to the current pandemic. For example, hospitals in Saudi Arabia have already established triage units for respiratory diseases equipped with negative-pressure ventilation systems to limit infection (8). The Kingdom was also prepared to implement strict containment measures that ensured limited social movement, while other countries hesitated to take such measures. The Saudi public largely adhered to these measures; however, the toll of this restriction impacted the finances and psychological well-being of many citizens (10). When successive COVID-19 waves occurred, the government implemented less restrictive NPIs guidelines; adherence became voluntary. This study aims to understand why residents of Saudi Arabia were adherent or non-adherent to voluntary NPIs recommendations.

As past pandemics have illustrated and COVID-19 emphasized, the response to a viral outbreak must be collaborative between governments, healthcare organizations, businesses, media outlets, and community members (11). The collective response forms a complex and interacting system that places individuals and their behaviors at the nexus of the system (6). Therefore, using behavioral sciences to understand the psychological, social, and environmental factors is essential to developing health promotion interventions, drafting health-oriented policies, and shaping effective health communication campaigns (6). During the partial curfew period, researchers found that most people in Saudi Arabia had optimistic attitudes about the threat of COVID-19 and overall good adherence to avoiding social gatherings and washing hands (12). This study adds to the Al-Hanawi et al. (12) study by studying the adoption of NPIs recommendations within the theoretical lens of the Social Cognitive Theory.

Studies of behavioral responses to emerging infectious diseases have found success in applying expectancy-value theories such as the Health Belief Model and the Theory of Planned Behavior (13) to understanding individual risk behaviors (14–17). However, these theories downplay the importance of social and structural influences on adherence to prescribed guidelines. For example, the Health Belief model offers little explanation of why subgroups of a population vary in their adherence to health behavioral recommendations (18).

Individual behavior is influenced by cognitive and affective factors and by sociocultural factors such as social norms. Other factors, such as resource availability and effective health messaging, play an important role (19). Studies show that risk perceptions and preparedness behaviors vary across countries, even after controlling for educational status, age, and employment status (20). This result suggests that social-cultural influences on behavior play an important role in behavior change. Prati et al. (21) used a social-cognitive framework to study adherence during the H1N1 pandemic in 2009. Social factors such as exposure to media campaigns were predictive of pandemic protective behaviors without mediating the respondents’ affective responses, whereas the cognitive factors were mediated by affect. Wong and Jensen (19) studied adherence to COVID-19 guidelines and emphasized the need to consider social and cultural factors when examining how individuals take action to reduce their health risks. While media campaigns encouraged behavior change, they did little to change emotional responses to the risk; however, knowledge, understanding, and self-efficacy were improved by the campaigns.

In some governance systems, such as those in China, Russia, and Hungary, decision-making authority is more centralized. Saudi Arabia also follows a model where leadership is concentrated within a central authority. In contrast, countries like the United States, South Korea, and Australia have more decentralized structures, with power shared across federal, state, and local levels. Culturally, societies with more centralized are more accustomed to top-down decision-making. In such settings, there is often a greater emphasis on unity, trust in leadership, and alignment with government guidance (22, 23). On the other hand, citizens living in decentralized structures are generally more accustomed to differences of opinion between public officials and domain experts. In contexts where trust in government is lower, individuals may be more likely to express discontent, act independently of official recommendations (24, 25), and report lower confidence in leadership (26). Recently, Choi et al. (27) compared adherence to pandemic recommendations across six countries (USA, Italy, Korea, Australia, Finland and Sweden) and showed higher adherence and behavioral intentions was associated with higher trust in government, but adherence was lowered when citizens were less accustomed to governments with higher levels of power difference. Predicting adherence to health recommendations, thus, is complicated by style of government, cultural and individual orientation toward leaders, communication clarity, trust, and myriad other factors. The complexity of findings across the studies cited here demonstrate the need for research conducted in countries where styles of government differ widely.

Investigating non-pharmaceutical interventions (NPIs) within a Saudi population offers a unique perspective, given the country’s experience with a major coronavirus outbreak prior to COVID-19. In 2012, during the emergence of Middle East Respiratory Syndrome (MERS) in Saudi Arabia, NPIs were the primary defense against a highly fatal virus (9). This context makes it particularly important to examine adherence to NPIs in Saudi Arabia, as it sheds light on how prior epidemic experiences may shape public behavior during subsequent health crises. Specifically, the social cognitive model used in our study includes constructs such as outcome expectancy and knowledge, both of which can be influenced by previous experiences. This study explores the influence of social, cultural, and cognitive factors on adopting COVID-19 non-pharmaceutical interventions and addresses the research question: Which demographic, cognitive, or socio-environmental factors are more predictive of adherence to COVID-19 NPIs among the Saudi population?

## 2 Methods

### 2.1 Participants and procedures

This study follows a non-probability convenience sampling strategy, where a web-based survey was used to recruit study participants. Prior studies in Saudi Arabia have shown potential participants to be receptive to internet-based studies (12). This study recruited participants and distributed an online survey to King Saud bin Abdulaziz University for Health Sciences (KSAU-HS) staff and students via electronic bulletin boards, emailed fliers, and select social media groups. Participants over 18 years of age who lived in Al-Ahsa region were recruited. Participants were encouraged to invite others on their social networks. The Cochran’s formula for calculating sample size for survey research was used to calculate a sample size of 384 (28).

### 2.2 Measurements

To measure adherence to COVID-19 non-pharmaceutical behavioral recommendations, a 59-item survey based on Social Cognitive Theory was developed. All survey items were reviewed by subject matter to ensure theoretical constructs are properly covered. The survey was initially developed in English and then translated into Arabic. To test the translation, an Arabic-speaking PhD student not associated with the study translated the survey back into English. Additionally, two rounds of pilot testing with two distinct participant groups were conducted. Changes were implemented as needed, and the final survey was retested for linguistic accuracy. The survey was open from March 02, 2021, to April 4, 2021. SurveyMonkey was used to disseminate the survey to the study participants.

In addition to the information described below, the survey elicited sociodemographic information, including age, gender, marital status, education, occupation, nationality, place of residence, number of household members, average monthly income, vaccination status, and the presence of COVID-19 health risk factors.

#### 2.2.1 Personal cognitive factors

Self-efficacy beliefs of adopting COVID-19 NPIs, outcome expectancies of adherence to NPIs, and knowledge of COVID-19 were assessed. A seven Likert-type scale items was developed to measure the perceived ability to adopt COVID-19 NPIs; for example, one question is: “I can maintain my social distance (1.5 m) outside my residence.” Options were presented as “1 = strongly disagree to 5 = strongly agree.” An aggregate variable was created by summing the seven items, with scores ranging from 7 to 35, with higher scores indicating higher perceived self-efficacy levels. The aggregate variable showed good internal reliability [Cronbach’s Alpha = 0.83; Mohajan (29)].

To assess outcome expectancies, a five Likert-type questions were created asking participants about the perceived outcomes of adopting COVID-19 NPIs. A sample item is “I believe that following COVID-19 safety measures will be beneficial to my health.” Scale items ranged from “1 = strongly disagree to 5 = strongly agree.” Aggregate scores ranged from five to 25, with higher scores indicating higher perceived positive outcomes of adherence to COVID-19 NPIs behaviors. The aggregate variable had a Cronbach’s Alpha of 0.76, indicating satisfactory internal reliability.

Knowledge was assessed about COVID-19 symptoms, transmission routes, and understanding of the effectiveness of NPIs behaviors in preventing infection. Knowledge items were developed from the available information from the CDC, the WHO, and the Saudi Arabian Ministry of Health; final validation of the knowledge items was done by comparing them to the Understanding America Study website (30). Knowledge items were presented as true or false. Incorrect or “do not know” answers were coded as zero, and correct answers were coded as one. Total scores ranged from zero to 13, with higher scores indicating higher COVID-19 knowledge.

#### 2.2.2 Socioenvironmental factors

Three constructs to evaluate socio-environmental factors were used: participants’ perceptions of the social norms of adopting COVID-19 NPIs, media exposure, and participants’ perception of what observational learning had occurred. Social norms were measured with four questions relating to how frequently they perceived Influential others as performing COVID-19 NPIs. A sample item is “Please indicate how much do you think the people close to you accept and practice social distancing.” The five-point Likert scale ranged from “1 = never to 5 = always.” Total scores ranged from four to 20, with higher scores indicating COVID-19 NPIs to be more socially acceptable. The resulting Cronbach’s alpha coefficient was 0.88, indicating satisfactory internal reliability.

Media exposure was a single item factor asking the participant how they perceived their media exposure to COVID-19-related information: “Do you think you are receiving the necessary COVID-19 related information you need to make a decision about your health?” Choice options were “yes,” “no,” or “I do not know.” Observational learning was a single item factor asking the participant: “I had to learn new skills to properly perform the recommended precautionary measures (e.g., face covering, hand washing).” Choice options were “yes,” “no,” or “I do not know.”

#### 2.2.3 Adherence to NPIs behaviors

The main dependent variable was the self-reported frequency of the performance of NPIs: mask-wearing, hand washing, social distancing, and quarantining/isolating. A sample item was “Please indicate how frequently you wear a face mask when you leave your residence.” The five-point Likert scale ranged from “1 = never to 5 = always.” Total scores ranged from four to 20, with higher scores indicating more adherence to COVID-19 NPIs. The aggregate variable had a Cronbach’s alpha coefficient of 0.80, indicating satisfactory internal reliability.

### 2.3 Analytical analysis

Parametric and non-parametric analyses were used where appropriate. Cross-tabulations and other descriptive reports were generated. Correlation, t-tests, and analysis of variance (ANOVA) were used to determine group differences in the dependent variable. A hierarchical multiple regression model was built to explore the influence of the social-cognitive and demographic factors on the adoption of NPIs. Model building was ordered based on demographics, personal cognitive factors, and socioenvironmental factors. Analyses were done using SPSS software (IBM SPSS Statistics for Windows, Version 27), and type 1 errors were limited to alpha = 0.05.

### 2.4 Ethics approval statement

This study and all its related procedures involving human participants followed national and international research committee ethical standards. The design and execution of this study were evaluated, approved, and exempted from obtaining signed consent by the institutional review board of the University of Louisville (Ref# 713658). The research committee at King Saud Bin Abdulaziz University for Health Sciences reviewed the ethical approval by the University of Louisville and deemed it to be sufficient for their approval.

## 3 Results

### 3.1 Characteristics of the study respondents

After excluding 38 participants who lived outside the study catchment area, the sample included 368 participants. The sample was mostly male (62%); 80% lived in urban Al-Ahsa, 25% were students, and around 68% reported the Ministry of Health as their main source for COVID-19 information. Additionally, 44% of the study sample received at least one dose of the COVID-19 vaccine. In Tables 1, 2, 3, study participants’ social and demographic characteristics are presented, along with the mean scores of the main variables.

**Table 1:** Summary data of the social and demographic characteristics of study participants.

Variable	Mean	SD	*N*	%
NPIs level	16.61	3.78		
Knowledge	10.00	3.21		
Self-efficacy	30.73	3.91		
Social norms	15.46	4.34		
Outcome Expectancies	21.28	3.36		
Age	32.14	11.70		
Sex				
Female			119	37.3
Male			200	62.3
Residence				
Urban Al-Ahsa			269	85.3
Rural Al-Ahsa			51	14.7
Single			160	50.2
Marital status				
Married			150	47.0
Occupation				
Health			38	11.9
Education			71	22.3
Student			78	24.5
Unemployed			35	11
Other			97	30.4
Education				
Middle school			5	1.6
High school			86	27.0
2 Year diploma			39	12.2
Bachelor’s degree			180	56.4
Graduate degree			9	2.8
Nationality				
Saudi			311	97.5
Non-Saudi			8	2.5
Household members				
Less than 6			139	43.8
Six and more			178	56.2
Presence of chronic diseases				
Yes			21	6.6
No			295	93.4
Monthly income				
Less than 3,000 SAR			66	20.7
3,000 to 8,999 SAR			71	22.3
9,000 to 14,999 SAR			81	25.4
15,000 to 29,999 SAR			34	10.7
30,000 SAR and above			10	3.1
COVID-19 testing				
Never tested			116	31.5
Tested, negative result			180	48.9
Tested, positive result			61	16.6
Vaccinated against COVID-19				
Yes			160	44.2
No			202	55.8
COVID-19 information				
Ministry of Health			249	67.7
Official social media accounts			69	18.8
Unofficial social media accounts			19	5.2
Doctor			9	2.4
Media exposure				
Sufficient			219	77.9
Insufficient			62	22.1
Learned New skills to protect against COVID-19?				
Yes			172	64.9
No			93	35.1

**Table 2:** Mean scores of NPIs adherence level and social norms among social and demographic groups.

Variable			NPIs score			Social norms score		
	*N*	%	*M* (CI)	SD	*p*	*M* (CI)	SD	*p*
Sex								
Female	119	37.3	17.04 (16.3–17.7)	3.86	0.03	15.56	4.09	
Male	200	62.3	16.29 (15.7–16.9)	4.12		15.40	4.40	
Residence								
Urban Al-Ahsa	269	85.3	16.27 (15.8–16.8)	4.25	0.03	15.44	4.45	
Rural Al-Ahsa	51	14.7	17.69 (17.2–18.2)	1.85		15.54	3.19	
Education								
High School	86	27.0	16.04	4.17		15.31	4.36	
2 Year Diploma	39	12.2	17.25	3.63		16.33	4.24	
Bachelor’s Degree	180	56.4	16.34	4.15		15.33	4.41	
Household members								
Less than 6	139	43.8	16.13	4.30		15.40	4.24	
Six and more	178	56.2	16.74	3.80		15.50	4.36	
Monthly income								
Less than 3,000 SAR	66	20.7	16.49	3.56		15.82	4.06	
3,000 to 8,999 SAR	71	22.3	15.31	5.05		14.33	5.42	
9,000 to 14,999 SAR	81	25.4	16.58	3.67		15.75	3.66	
15,000 to 29,999 SAR	34	10.7	16.43	3.93		15.00	3.72	
30,000 SAR and above	10	3.1	16.75	3.56		14.88	3.56	
COVID-19 testing								
Never tested	116	31.5	15.94	4.67		15.13	4.83	
Tested, negative result	180	48.9	16.52	3.52		15.62	3.99	
Tested, positive result	61	16.6	16.37	4.73		15.60	4.61	
Vaccinated against COVID-19								
Yes	160	44.2	16.34	4.20		15.40	4.58	
No	202	55.8	16.58	3.89		15.51	3.99	
COVID-19 information								
Ministry of Health	249	67.7	16.82	3.95		15.76	4.15	
Official social media	69	18.8	15.68	3.91		15.24	4.28	
Unofficial social media	19	5.2	16.88	2.30		13.75	5.92	
Media exposure								
Sufficient	219	77.9	16.90 (16.4–17.4)	3.68	0.02	15.85 (15.3–16.4)	4.06	0.04
Insufficient	62	22.1	14.98 (13.8–16.2)	4.82		14.09 (12.9–15.3)	4.82	
Learned new skills								
Yes	172	64.9	16.02 (15.3–16.7)	4.54	0.02	15.09	4.62	
No	93	35.1	17.30 (16.8–17.8)	2.70		16.15	3.53	

**Table 3:** Mean scores of cognitive predictor variables among social and demographic groups.

Variable			Knowledge		Self-efficacy			Outcome expectancies			
	*N*	%	M (CI)	SD	*p*	*M* (CI)	SD	*p*	*M*	SD	*p*
Sex											
Female	119	37.3	10.94	1.73		31.21	3.62		21.53	3.52	
Male	200	62.3	10.88	1.77		30.44	4.03		21.14	3.52	
Residence											
Urban Al-Ahsa	269	85.3	10.83	1.82		30.79	3.97		21.45	3.45	
Rural Al-Ahsa	51	14.7	11.35	1.13		29.98	3.63		20.50	2.96	
Education											
High school	86	27.0	10.63	2.11		30.47	4.16		20.83	3.46	
2 Year diploma	39	12.2	10.96	1.40		32.00	3.55		21.13	2.88	
Bachelor’s degree	180	56.4	11.00	1.63		30.55	3.79		21.50	3.56	
Household members											
Less than 6	139	43.8	11.05	1.73		30.47	4.19		21.27	3.52	
Six and more	178	56.2	10.77	1.76		30.90	3.70		21.36	3.31	
Monthly income											
Less than 3,000 SAR	66	20.7	10.44	2.00		31.26	3.70		20.85	4.22	
3,000 to 8,999 SAR	71	22.3	11.10	1.45		30.96	3.64		21.76	2.96	
9,000 to 14,999 SAR	81	25.4	10.98	1.59		30.78	3.69		21.35	2.81	
15,000 to 29,999 SAR	34	10.7	10.95	1.43		29.82	4.52		21.43	3.41	
30,000 SAR and above	10	3.1	11.75	1.04		29.75	3.77		21.63	2.26	
COVID-19 testing											
Never tested	116	31.5	10.94	2.00		30.84	3.98		20.38	4.02	
Tested negative	180	48.9	11.01	1.45		31.01	3.73		21.70	3.09	
Tested positive	61	16.6	10.57	2.08		30.06	4.02		21.77	2.87	
Vaccinated against COVID-19											
Yes	160	44.2	10.84	1.69		30.94	3.97		21.83 (21.4–22.3)	2.88	0.01
No	202	55.8	10.95	1.81		30.50	3.83		20.84 (20.3–21.4)	3.77	
COVID-19 information											
Ministry of Health	249	67.7	11.09	1.60		31.13 (30.6–31.6)	3.76	0.02	21.64 (21.3–22)	2.98	0.05
Official social media	69	18.8	10.73	1.76		29.90 (29–30.8)	3.90		20.68 (19.6–21.7)	4.43	
Unofficial social media	19	5.2	9.63	2.50		28.75 (27.1–30.4)	3.67		20.62 (19.3–22)	2.97	
Media exposure											
Sufficient	219	77.9	10.90	1.79		31.63 (31.2–32.1)	3.30	<0.001	21.56	3.21	
Insufficient	62	22.1	10.89	1.63		29.20 (28.1–30.3)	4.55		20.50	3.92	
Learned new skills											
Yes	172	64.9	10.73 (10.5–11)	1.81	0.03	30.70	4.03		21.68 (21.2–22.2)	3.31	0.05
No	93	35.1	11.21 (10.9–11.5)	1.59		30.72	3.68		20.64 (19.9–21.3)	3.48	

### 3.2 Adherence to non-pharmaceutical interventions

The overall mean frequency of NPIs behaviors was 16.61 (*SD* = 3.78). Females reported higher level of NPIs practice than males (*p* = 0.03); those living in rural Al-Ahsa were more adherent to NPIs behaviors than those in urban Al-Ahsa (*p* = 0.03). Those who reported receiving enough information about COVID-19 from the media reported practicing NPIs behaviors more than those who thought they did not receive enough information (*p* = 0.02). Those who reported the need to learn new skills to perform NPIs behaviors engaged in fewer NPIs behaviors than those who already knew how to perform the behaviors (*p* = 0.04). No statistically significant differences were found in adherence to non-pharmaceutical interventions (NPIs) based on participants’ education level, number of household members, monthly income, COVID-19 testing history, or vaccination status (Table 4).

**Table 4:** Pearson’s correlation matrix for behavioral, cognitive, and socioenvironmental factors.

Variable	1	2	3	4	5
1. NPIs	–				
2. Social norms	0.73**	–			
3. Outcome expectancies	0.06	0.06	–		
4. Self-efficacy	0.25**	0.21**	0.49**	–	
5. Knowledge	0.08	0.08	0.04	0.1	–

NPIs, non-pharmaceutical interventions; **p < 0.001.

### 3.3 Social norms

The mean social norms score was 15.46 (*SD* = 4.34); significant difference in perceived social norms was found between participants who received enough COVID-19 information from the media and those who did not (*p* = 0.04). No other group-level differences in social norms were observed (Table 4). Non-pharmaceutical interventions were strongly correlated with social norms (*r* = 0.73, *p* < 0.001).

### 3.4 Cognitive factors

Overall mean knowledge was 10 out of 13 (*SD* = 3.21). Independent samples t-test revealed that those who did not have to learn new NPIs skills had higher knowledge scores than those who did (*p* = 0.03). Overall mean self-efficacy was 30.73 (*SD* = 3.91). Self-efficacy differed as a function of media exposure. The highest self-efficacy scores were found among those who considered the Saudi Ministry of Health as their main source of information (*p* = 0.02) and those who believed they were receiving the adequate amount of information (*p* = <0.001). As Table 4 shows, self-efficacy, and outcome expectancies were moderately correlated (*r* = 0.49, *p* < 0.001). The mean outcome expectancies level among the study sample was 21.28 out of 25 (*SD* = 3.36); those who received the COVID-19 vaccine had significantly higher outcome expectancies levels than those who answered “No” to the vaccination question (*p* = 0.01). No other group differences were observed (Table 5).

**Table 5:** Hierarchical regression analysis summary for NPIs increase on demographics, cognitive factors, and socioenvironmental factors.

Model and predictor variable	*β*	*t*	*Sig.*	*R* ^2^	*∆R* ^2^	*F*	*df*
**Model 1 – Demographics**			0.442	0.014	–	0.901	(3, 187)
Age	0.009	1.344					
Sex	0.065	0.442					
Vaccine	−0.111	−0.780					
**Model 2 – Add cognitive factors**			0.116	0.045	0.031	1.46	(3, 184)
Self-efficacy	0.317	2.252					
Outcome expectancies	−0.164	−1.484					
Knowledge	0.027	0.694					
**Model 3- Add socioenvironmental factors**			<0.001**	0.586	0.541	28.44	(3, 181)
Social norms	0.663	14.884					
Media	−0.103	−1.060					
Observational learning	0.109	0.969					

NPIs, non-pharmaceutical interventions; Δ*R*^2^, change in *R*^2^; F, F-statistic; **p < 0.001.

### 3.5 Social and cognitive influences on non-pharmaceutical interventions

**Figure 1:**
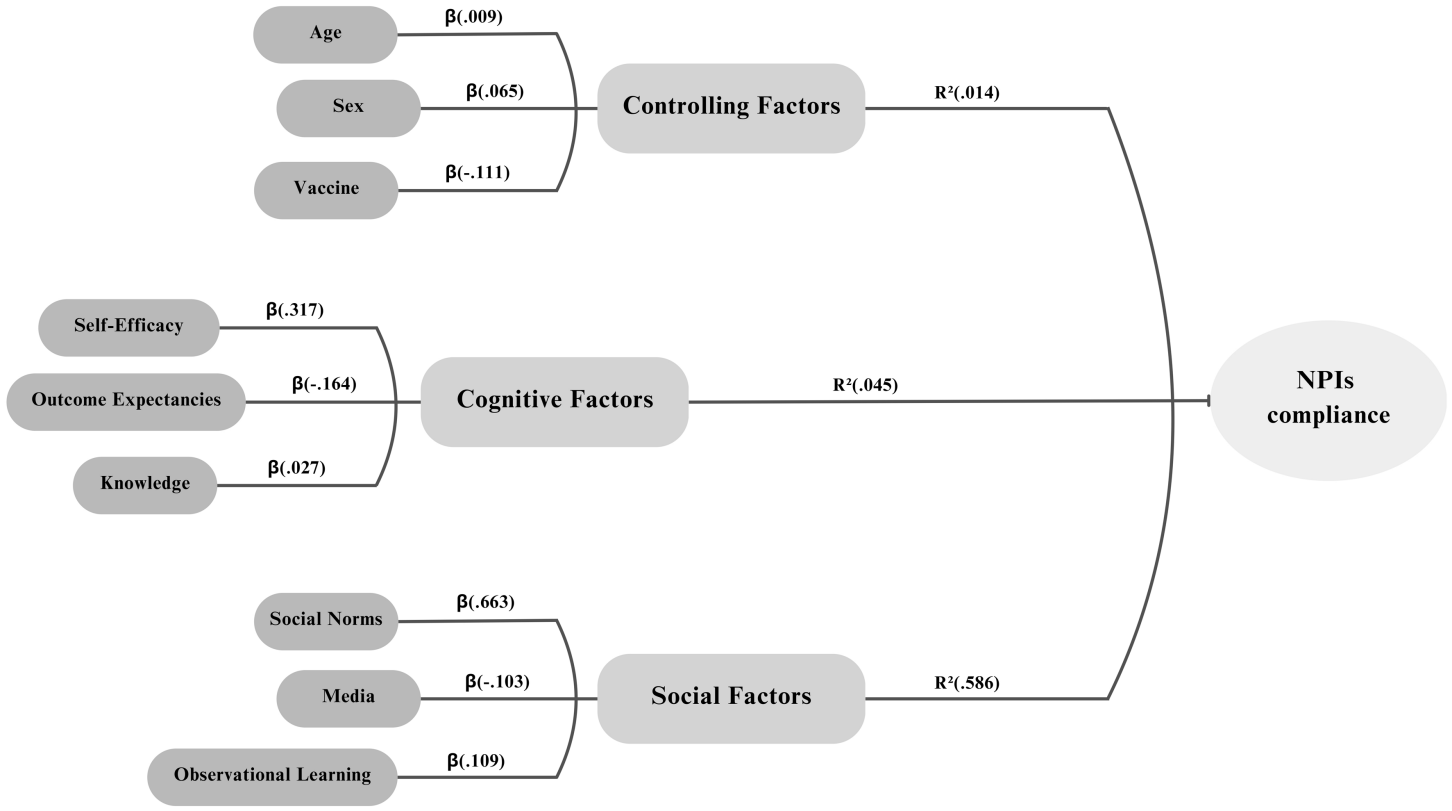
Relationship between controlling, cognitive, and social factors and adherence to non-pharmaceutical interventions (NPIs).

A hierarchical linear regression model was built to assess the factors that influenced NPIs adherence. Demographic factors were first entered into the model. These factors, which included sex, age, and vaccination status, only accounted for 1 % of the variability in NPIs behaviors (*R*^2^ = 0.01, *p* = 0.44.) The next block, cognitive factors, included self-efficacy, outcome expectancies, and knowledge. This block accounted for 4.5% of the variation in NPIs adherence (*R*^2^ = 0.03, *p* = 0.11). Among the cognitive factors, self-efficacy was most influential (*p* < 0.01). The third block, socio-environmental factors, which included social norm, media exposure, and observational learning, explained an additional 58.6% of the variation in NPIs adherence, and the change in *R*^2^ was significant, (*p* < 0.001). Among the socio-environmental factors, social norms were the most influential (*p* < 0.001) (Figure 1).

## 4 Discussion

The present study is the first to apply a fully integrated social, cognitive, and environmental model to understanding NPIs within the Saudi population. Previous studies have considered social-cognitive factors to understand single behaviors (31), separately evaluated social and socioenvironmental constructs (32), measured psychological factors (33), or reviewed multiple behavioral models and concluded the usefulness of a social cognitive model (18). The current study adds to the literature by exploring social cognitive factors in a sample of urban and rural Saudi Arabians. The timing of this study is important. Data collection for this study was in the early stages of vaccine rollout and when the Saudi Ministry of Health loosened its most stringent restrictions (34). Accordingly, and due to the similarity in vaccine hesitancy/refusal and NPIs non-adherence, vaccine status were controlled for in the hierarchical regression model (35). Understanding behavioral decision-making in Saudi Arabia is important as this country has had a recent and deadly experience with another coronavirus outbreak; cases and fatalities from MERS were highest in Saudi Arabia (9). Hence, the Saudi public are receptive to restrictive measures such as curfews, as long as they provide protection from infection (36).

The regression model showed that social norms are an important indicator of NPIs adherence and the most influential among all social factors. Social norms have consistently shown a strong relationship with NPIs at different time points of the COVID-19 pandemic, even when the criteria for NPI’s was slightly different. In early studies of the COVID pandemic, researchers found social norms were highly predictive of behaviors such as avoiding gatherings in public places, forgoing handshaking, and disinfecting surfaces around the house (37). Other researchers have found that decreased levels of perceived social norms of staying at home predicted low adherence levels to staying at home (38). Moreover, similar associations between social norms and NPIs have been found in other countries (39). These previous results, coupled with ours, suggest the importance of social norms in influencing NPIs behaviors even in countries where the health security of the population is prioritized over individual freedoms (40).

Social norms can only influence behavior change to the extent to which these norms are observable (18). Establishing the relationship between social norms and NPIs in Saudi society is the first step in building more rigorous and effective public health interventions. The findings support public health agencies’ efforts to normalize the use of NPIs. This paper conceptualize that social norms differ between subgroups of the Saudi community and that understanding these differences is crucial to building effective health campaigns to create a more equitable pandemic response.

Cross-cultural analysis highlights distinct patterns in the ways that societies perceive health risks and adopt behavioral responses. This held true during the pandemic and recent research has documented how cultural norms influenced COVID-19 responses across nations. For example, East Asian communities are influenced by collective responsibility which supports adoption of behaviors for the protection of the broader community as well as the individual (41). Latin America countries with collectivist cultures, such as Brazil, also had high adherence with the adoption of behaviors to mitigate the spread of COVID-19 (42). In contrast, in Western societies such as the U. S., health decisions are often guided by perceived personal benefit which is often prioritized over the collective benefits for the community. Additionally, mask-wearing was associated with political orientation with conservatives being more likely to endorse the message that mask-wearing is ineffective and may convey a message of weakness of the wearer (43). During the pandemic, there was more resistance to adoption of mask-wearing and common complaints were related to needs of the individual (44). Recognizing the influence of culture and social norms on the adoption of health behaviors is essential for developing health communication messaging that will resonate within communities.

Consistent with past literature, being a female was reported to be a significant predictor of adherence with NPIs (39, 45). This result may be better understood in light of previous findings that Saudi women often report better medication adherence, greater health knowledge, and a more positive attitude toward health recommendations (46–48). Future studies should explore the sociocultural factors that may explain these gender differences. Equally important, the study sample showed a low level of risk perception while displaying a high level of NPIs adherence. In a study of medical students in Saudi Arabia, participants showed moderate levels of risk perception (49). The low and moderate levels of risk perception among the Saudi samples raise the question of the quality of risk communication in the Kingdom. This finding supports the need to develop a framework for communicating risk during pandemics to increase NPIs adherence. The predominance of male participants reflects common sampling patterns in Saudi Arabia, where cultural and logistical barriers often limit female participation in research (50). Additionally, the relatively high vaccination rate among respondents may be explained by the inclusion of individuals with a health sciences background, as they are more inclined to trust health authorities and adhere to public health recommendations. Given these demographic and attitudinal factors, the findings should be interpreted with appropriate caution.

In the study sample, 68% of participants reported that the Ministry of Health was their main media source. The reliance on the Ministry of Health demonstrates the outsized influence a single source of information can have on risk perceptions. In other countries, where decentralized media outlets and social media are prominent, the influence of governmental health agencies likely differs. In decentralized media environments, it is often difficult to deliver a consistent health message or clearly trace the impact back to a single source (51). In contrast, Saudi Arabia’s centralized media structure has enabled the country to establish public trust and effectively mobilize the population toward unified national objectives during times of crisis, such as the COVID-19 pandemic (52).

Exploring cognitive and social factors is valuable to understanding why people practice NPIs differently. Measuring self-efficacy levels was important in two aspects. First, it allowed us to add the levels of self-efficacy among a Saudi sample to the broader global self-efficacy literature, where a similarly strong positive correlation between self-efficacy and NPIs was found (53). Second, self-efficacy levels were assessed in this study based on two important indicators: the source and sufficiency of COVID-19 information. Sufficient exposure to information from the leading health entity in the country led to significantly higher levels of self-efficacy. This finding is a significant testimony to the Ministry of Health’s concentrated efforts to educate the Saudi public about COVID-19 prevention (54). The Saudi Ministry of Health’s recent and effective management of the MERS pandemic may have contributed to public perceptions of its trustworthiness.

Vaccinated and unvaccinated individuals differed in outcome expectancies or perceived benefits levels. This finding is expected as the vaccinated had already adopted one recommended behavior – vaccination. It is safe to consider their positive outcome expectancies from getting vaccinated to be similar to adopting NPIs (35). The relationship between outcome expectancies and vaccine uptake has been well established; this study shed light on the relationship between NPIs’ outcome expectancies and vaccine uptake (55).

Future research should use longitudinal or intervention-based designs to better assess changes over time and causal relationships, as the current study captures only a single point in time based on participants’ self-reported perceptions and behaviors. Additionally, since the sample may not fully reflect the wider population and other relevant factors were not explored in depth, future studies should include more diverse groups and examine the sociocultural influences that may help explain the observed gender differences in NPIs adherence and attitudes.

## 5 Practical and policy implications

To strengthen public health communication in Saudi Arabia, the study findings support integrating socio-environmental influences into national strategies through social marketing approaches. Drawing from the 4 P’s of social marketing, we suggest designing campaigns that present NPIs as a socially expected product, emphasize the social cost of non-compliance as the “price,” utilize Twitter as a key “place,” and apply loss-framed messaging for promotion. Loss-framed messages have been shown to be effective in influencing preventive behaviors like vaccination uptake (56, 57). Given Saudi Arabia’s highly collectivist culture, where behaviors are often shaped by concern for social cohesion, this approach is particularly relevant (58). Studies confirm that Saudis are highly active on social media, especially Twitter (59, 60), and prior analysis of the Ministry of Health’s tweets during the pandemic shows strong public engagement when messages promote COVID-19 prevention (61). By leveraging these platforms to normalize protective behaviors and highlight existing social compliance, communication efforts can shift from purely educational to socially persuasive (62), aligning public behavior with perceived social norms and increasing the likelihood of community-wide adoption (63).

In addition to messaging strategies, the results support a more targeted approach to public health interventions. While this study was not designed to explain the root causes of variation in NPIs adherence, it does highlight the value of audience segmentation. Based on demographics or behavioral patterns, the Ministry of Health can use findings from this study to identify less compliant segments and tailor communication efforts accordingly (64, 65). Specifically, groups with lower health knowledge or risk perception should receive more focused messaging. Beyond communication, the study findings have implications for policy. Building on earlier preparedness efforts such as the establishment of the Saudi CDC and National Health Laboratory (66, 67), the next step should involve resilience-based strategies that enhance collective efficacy. Promoting strong social norms through policy and digital communication can foster a shared sense of responsibility, especially within a collectivist society like Saudi Arabia.

## 6 Conclusion

The study investigated NPIs among a Saudi sample through the lens of a Social Cognitive Model. An online survey was distributed to measure the sample’s overall NPIs adherence and which factors, personal-cognitive or socioenvironmental, are the most influential in the participants’ adherence to NPIs. The findings highlight the usefulness of a social cognitive model in predicting NPIs. The study results suggest that social factors, especially social norms, significantly influence the adoption of NPIs. Hence, social factors should be considered when developing public health campaigns against COVID-19 and future infectious diseases.

## Data Availability

The raw data supporting the conclusions of this article will be made available by the authors, without undue reservation.
